# Chemical screening links disulfiram with cardiac protection after ischemic injury

**DOI:** 10.1186/s13619-023-00170-x

**Published:** 2023-07-19

**Authors:** Yuanyuan Chen, Jianyong Du, Lixia Zheng, Zihao Wang, Zongwang Zhang, Zhengyuan Wu, Xiaojun Zhu, Jing-Wei Xiong

**Affiliations:** 1 Beijing Key Laboratory of Cardiometabolic Molecular Medicine, Institute of Molecular Medicine, College of Future Technology, and State Key Laboratory of Natural and Biomimetic Drugs, Peking University, Beijing, 100871 China; 2 School of Health and Life Sciences, University of Health and Rehabilitation Sciences, Qingdao, 266071 China; 3 PKU-Nanjing Institute of Translational Medicine, Nanjing, 211800 China

**Keywords:** Small molecule, High-throughput chemical screening, Disulfiram, Gasdermin D, Ischemia/reperfusion injury, Rat cardiomyocytes

## Abstract

Ischemia–reperfusion injury occurs after reperfusion treatment for patients suffering myocardial infarction, however the underlying mechanisms are incompletely understood and effective pharmacological interventions are limited. Here, we report the identification and characterization of the FDA-approved drug disulfiram (DSF) as a cardioprotective compound. By applying high-throughput chemical screening, we found that DSF decreased H_2_O_2_-induced cardiomyocyte death by inhibiting Gasdermin D, but not ALDH1, in cardiomyocytes. Oral gavage of DSF decreased myocardial infarct size and improved heart function after myocardial ischemia–reperfusion injury in rats. Therefore, this work reveals DSF as a potential therapeutic compound for the treatment of ischemic heart disease.

**Supplementary Information:**

The online version contains supplementary material available at 10.1186/s13619-023-00170-x.

## Background

Ischemic heart disease is one of the leading causes of death worldwide (Buja 2005; Virani et al. 2020). Myocardial ischemia is a pathological feature characterized by an insufficient coronary blood supply with respect to the metabolic requirements of the myocardium. Over the years, the predominant therapy to reduce ischemic injury is a reperfusion strategy (Ibáñez et al. 2015). However, timely restoration of blood flow to the ischemic zone also results in myocardial damage, termed “ischemia/reperfusion (I/R) injury” (Black 2000). This injury mainly results from the reactive oxygen species (ROS) produced during reperfusion, which contribute to cell death by apoptosis, pyroptosis, autophagy, and necrosis (Spinale 2010) at the infarct and its adjacent zones.

Although the reperfusion strategy contributes to a beneficial reduction in mortality rates, the reduced left ventricular ejection fraction resulting from reperfusion injury is one of the principal causes of chronic heart failure (Callender et al. 2014). Extensive studies have attempted to intervene in this adverse post-infarction outcome (Yancy et al. 2013), but the clinical implementation of non-pharmacological therapies has been substantially limited due to the high economic costs and the limited availability of heart donors (LaPointe et al. 2011). Compared with the expensive devices and heart transplantation, pharmacological strategies with small molecules have advantages in acceptable expense, convenient administration, and adjustable intervention (Liu et al. 2016; Liang et al. 2021). Notably, many small molecules have been reported to ameliorate reperfusion injury and reduce infarct size. One of the promising molecules, cyclosporine-A, has been proposed to inhibit the opening of the mitochondrial permeability transition pore when confronted with ROS, and thus prompt cell viability (Piot et al. 2008). Metoprolol, one of the β-blockers, acts on circulating cells (neutrophils/platelets) to confer cardioprotective effects when administered before reperfusion (Garcia-Prieto et al. 2014). Abciximab, one of the glycoprotein IIb/IIIa inhibitors, potently inhibits platelets and platelet-leukocyte aggregates in I/R injury (Stone et al. 2012). Nevertheless, a long list of small molecules has failed to demonstrate beneficial effects in reducing reperfusion injury in the pre-clinical phase, commonly due to weak efficacy and unclear targets (Jones et al. 2015; Fernandez-Jimenez and Ibanez 2015).

Given that searching for small-molecule drugs to reduce reperfusion injury is urgently needed, we established a high-content chemical screening system for identifying small molecules that are able to repress the oxidative effect of H_2_O_2_ on neonatal rat ventricular myocytes (NRVMs). H_2_O_2_ is the most abundant form of ROS produced during I/R and is widely used to mimic the in vivo cardiac pathology of I/R (Lee et al. 2013). In particular, we identified an FDA-approved drug, disulfiram (DSF), which ameliorated H_2_O_2_-induced cardiomyocyte (CM) death and IR-induced necrosis/pyroptosis potentially via the inhibition of Gasdermin D (GSDMD). GSDMD can be cleaved into GSDMD-NT and GSDMD-CT domains, GSDMD-NT oligomerizes and perforates the plasma membrane to promote the secretion of and IL-1β and IL-18 in macrophages, leading to cell death (Kayagaki et al. 2015). Consistent with this, recent studies have reported that GSDMD deficiency significantly reduces I/R-induced myocardial injury (Shi et al. 2021) and the IR-induced inflammatory response in acute myocardial infarction (AMI) (Jiang et al. 2022). Together, DSF and GSDMD have great potential for developing therapeutic interventions for AMI in humans.

## Results

### High throughput chemical screening identifies DSF as protecting CMs from H_2_O_2_-induced injury

**Fig. 1:**
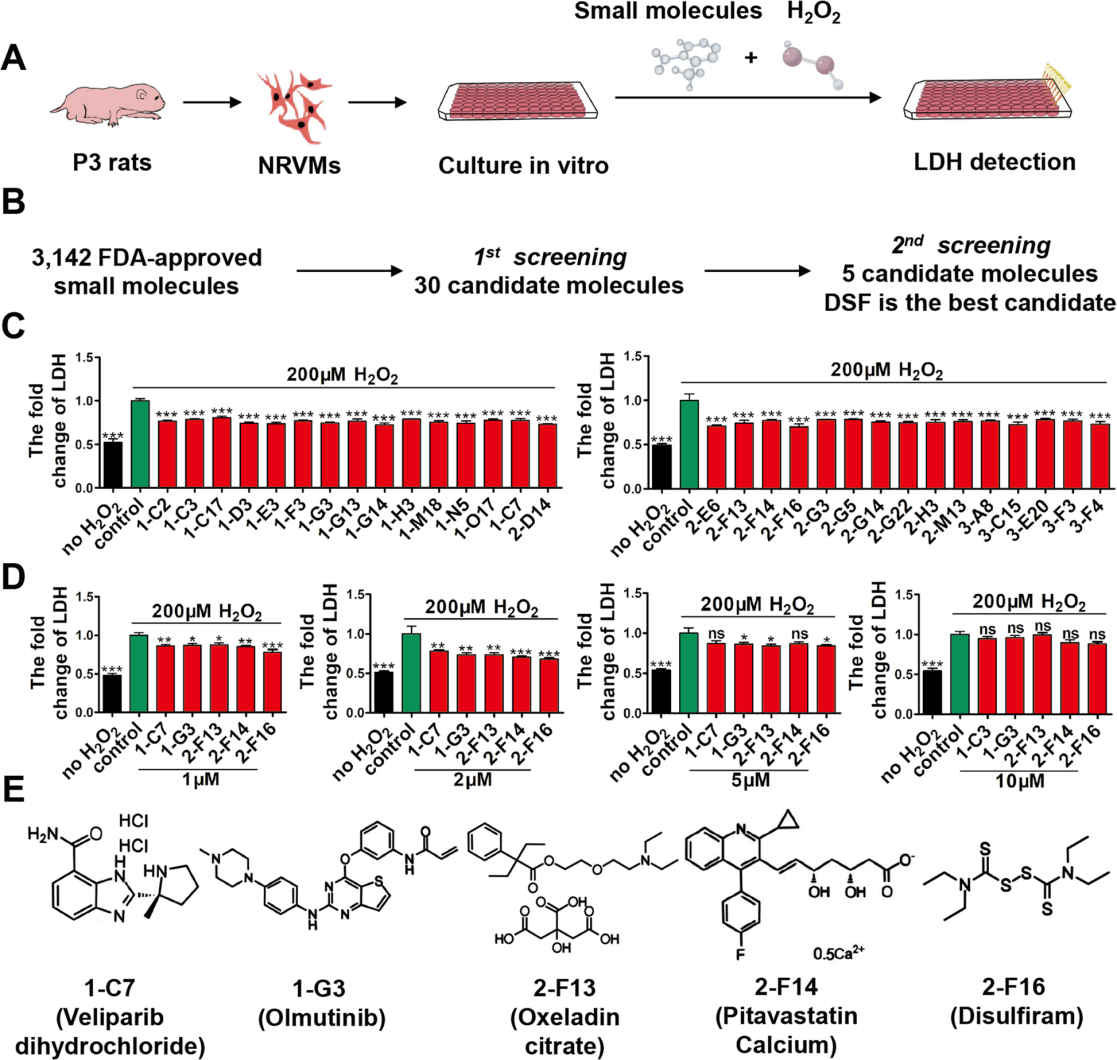
Chemical screening identifies small molecules that protect cardiomyocytes (CMs) against H_2_O_2_-induced oxidative injury. **A** Schematic of high-throughput screening of small molecules that suppress H_2_O_2_-induced injury of NRVMs by LDH assay. **B** Flowchart showing two-round chemical screening for small molecules that decrease H_2_O_2_-induced CM death. **C** The first-round chemical screening identifies 30 small molecules from 3,143 FDA-approved compounds based on LDH assays, where NRVMs are exposed to H_2_O_2_ (200 μM) in the presence of 2 mM compounds for 24 h. *n* = 3 per group. **D** The second-round screening confirms 5 candidate small molecules, each of which has a dose-dependent effect on LDH release in the presence of 1, 2, 5, or 10 μM compounds for 24 h. *n* = 4 per group. **E** The chemical structures of 5 candidate small molecules. 1-C7, Veliparib (dihydrochloride); 1-G3, Olmutinib; 2-F13, Oxeladin (citrate); 2-F14, Pitavastatin (Calcium); 2-F16, Disulfiram (DSF). *p < 0.1, **p < 0.01, ***p < 0.001; ns, no significant difference. One-way ANOVA with Dunnett’s test (**C**, **D**). Data are the mean ± s.e.m. (**C**, **D**)

To discover drug leads for ischemic heart disease, we established a high-throughput chemical screen for candidate compounds able to repress H_2_O_2_-induced CM death among 3,143 FDA-approved small molecules. We simultaneously treated NRVMs with H_2_O_2_ and 2 mM compounds for 24 h, and then applied a cell survival assay based on lactate dehydrogenase (LDH) release (Fig. 1A). In the first-round screening, NRVMs were incubated with each compound (2 μM) and H_2_O_2_ (200 μM) in 96-well plates for 24 h, and 30 candidates were found that effectively inhibited the release of LDH by CMs (Fig. 1B, C). In the second-round screening, we investigated the dose-dependent effect of the 30 compounds on the survival of NRVMs based on LDH release, and discovered 5 compounds that had protective effects on CMs at a low concentration (1 μM) and had no evident cytotoxicity at a high dose (5 μM) (Fig. 1D, E). Among the 5 candidates, we found that DSF had the strongest protective effect on CM injury.

**Fig. 2:**
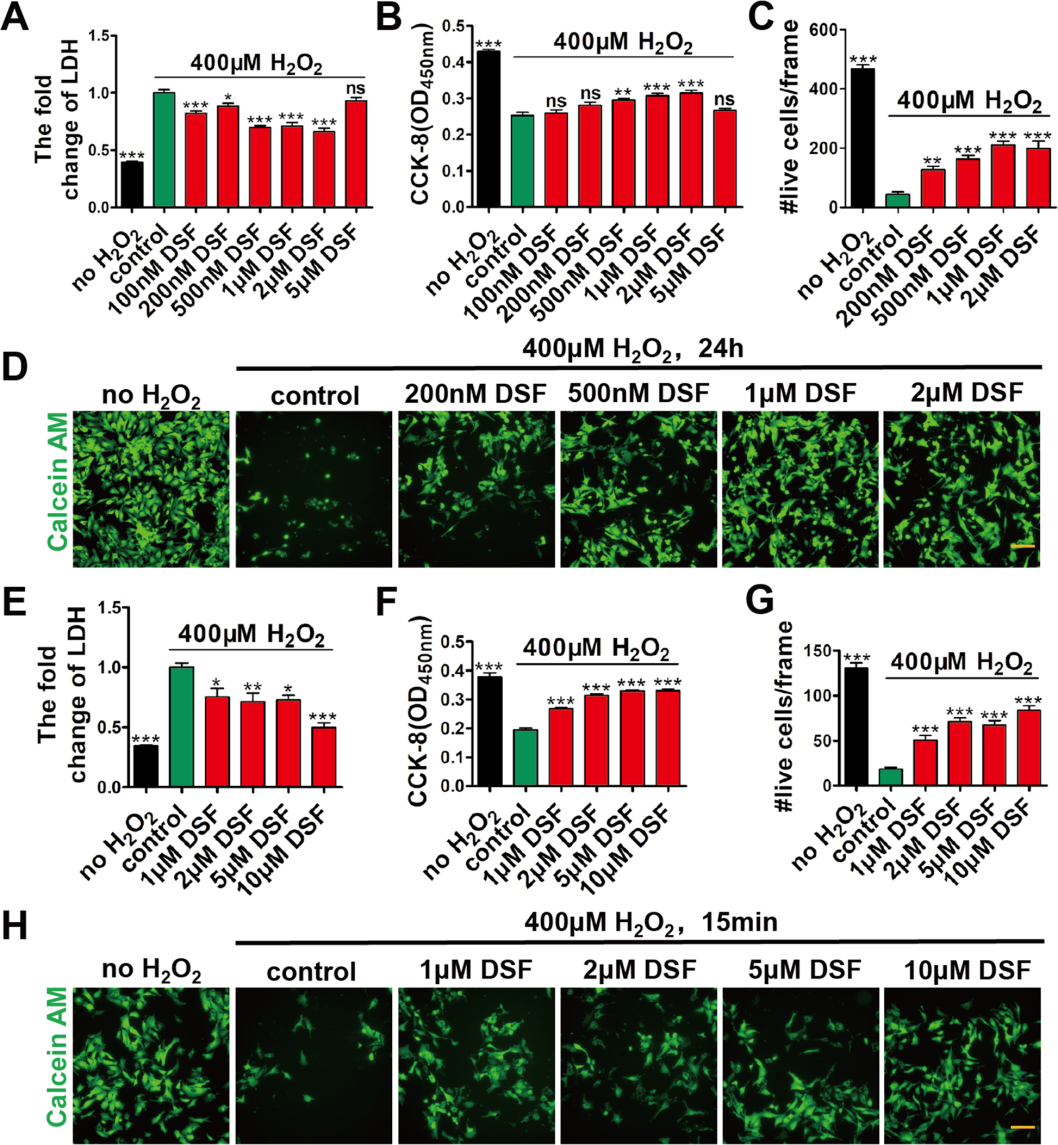
DSF treatment protects CMs from H_2_O_2_-induced injury in a dose-dependent manner. **A**-**D** Fold change of LDH release (**A**), CCK-8 detection (**B**), numbers of live CMs (**C**), and representative images of calcein AM staining (**D**) in NRVMs after exposure to H_2_O_2_ (400 μM) and different concentrations of DSF for 24 h. *n* = 6 (A, B), or *n* = 5 (**C**, **D**) per group. **E**–**H** Fold change of LDH release (**E**), CCK-8 detection (**F**), numbers of live CMs (**G**), and representative images of calcein AM staining (**H**) in NRVMs exposed to 400 μM H_2_O_2_ for 15 min, followed by treatment with different concentrations of DSF for 24 h. *n* = 6 (**E**, **F**), or 5 (**G**, **H**) per group. Scale bar, 100 μm (**D**, **H**). *p < 0.1, **p < 0.01, ***p < 0.001; ns, no significant difference. One-way ANOVA with Dunnett’s test (**A**-**C**, **E**–**G**). Data are the mean ± s.e.m. (**A**-**C**, **E**–**G**)

To further study the inhibitory effect of DSF on cardiomyocyte injury under oxidative stress, we performed assays on LDH release, CCK-8, and calcein AM staining in NRVMs that were treated with H_2_O_2_ in the presence of different concentrations of DSF for 24 h. Under the oxidative stress of H_2_O_2_ (200 μM), all groups with DSF treatment showed improved cell viability compared with the control group, especially in the groups with DSF from 0.5 to 2 μM (Fig. S1A–D). Consistent with this, when NRVMs were exposed to more harsh oxidative stress at 400 μM H_2_O_2_, DSF treatment (0.5, 1, and 2 μΜ) decreased LDH release (Fig. 2A), and increased CCK-8 (Fig. 2B) and the numbers of live CMs (Fig. 2C, D).

We next determined whether DSF treatment confers better beneficial effects when administered before oxidative stress damage. NRVMs were first treated with DSF for 24 h, and then exposed to H_2_O_2_ for 15 min in the absence of DSF. We found that, under H_2_O_2_ exposure (200 μM), all DSF treatment groups had reduced LDH release and increased CCK-8 in NRVMs (Fig. S1E, F). Notably, higher concentrations of DSF treatment (5 and 10 μM) prior to H_2_O_2_ exposure resulted in better protective effects on NRVMs compared with those via simultaneously applying both H_2_O_2_ and DSF. Likewise, when NRVMs were exposed to higher oxidative stress at 400 μM H_2_O_2_, all DSF treatment groups had reduced LDH release and increased CCK-8 and calcein AM staining compared with those in the control group (Fig. 2E–H), demonstrating that DSF pre-treatment dramatically ameliorated CM injury. Collectively, these data demonstrated that DSF treatment attenuates H_2_O_2_-induced CM damage and DSF pre-treatment has better protection in a dose-dependent manner.

### DSF protects CMs from H_2_O_2_-induced damage via inhibiting GSDMD

**Fig. 3:**
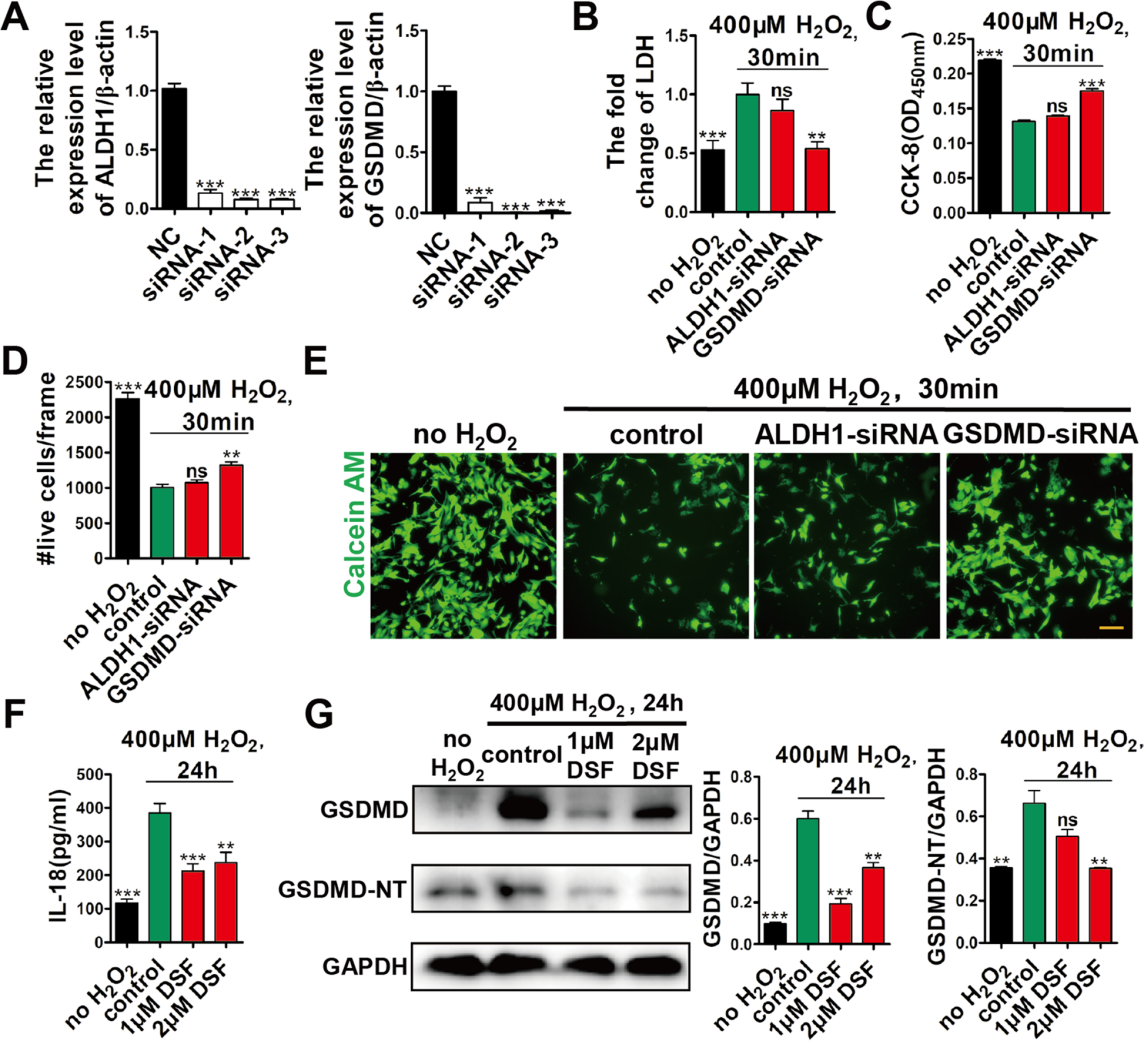
DSF protects CMs from H_2_O_2_-induced injury by inhibiting GSDMD but not ALDH1. **A** qRT-PCR showing siRNA knockdown efficiency targeting either ALDH1 or GSDMD (3 siRNA/gene) in NRVMs. *n* = 2 per group. **B**-**E** Fold change of LDH release (**B**), CCK-8 detection (**C**), numbers of live CMs (**D**), and representative images of calcein AM staining (**E**) in NRVMs in the presence or absence of ALDH1- or GSDMD-siRNA and then exposed to 400 μM H_2_O_2_ for 30 min. *n* = 4 (**B**, **C**) or 5 (**D**, **E**) per group. **F**, **G** ELISA results of IL-18 release (**F**) and western blot analysis of GSDMD and GSDMD-NT expression (**G**) in NRVMs after exposure to 400 μM H_2_O_2_ in the presence or absence of DSF for 24 h. *n* = 4 (**F**) or 2 (**G**) per group. Scale bar, 100 μm (**E**). **p < 0.01, ***p < 0.001; ns, no significant difference. One-way ANOVA with Dunnett’s test (**A**-**D**, **F**, **G**). Data are the mean ± s.e.m. **A**-**D**, **F**, **G**

It is known that DSF has two potential targets, acetaldehyde dehydrogenase (ALDH) and GSDMD (Johansson 1992). As a chemical inhibitor of ALDH, DSF was first used in the western world to treat alcoholism (Fuller and Richard 1986). Recently, DSF was found to inhibit GSDMD pore formation and subsequent pyroptosis (Hu et al. 2020). To address DSF targets in CMs, we applied small interfering RNAs (siRNAs) to knock down either ALDH1 or GSDMD (Fig. 3A), and then examined the effect of DSF on CM protection upon exposure to H_2_O_2_. Quantitative real-time PCR showed that either ALDH1 or GSDMD siRNAs effectively knocked down the targeted genes (Fig. 3A). After siRNA transfection, we exposed NRVMs to 400 μM H_2_O_2_ for 30 min, and found that GSDMD siRNA, but not ALDH1 siRNA, decreased the LDH release, and increased CCK-8 and calcein AM staining (Fig. 3B–E). Moreover, after NRVMs were exposed to both H_2_O_2_ (400 μM) and DSF for 24 h, DSF significantly decreased IL-18 release and downregulated the expression levels of GSDMD and GSDMD-NT compared with the control group (Fig. 3F, G). Thus, these data suggested that DSF protects CMs from H_2_O_2_-induced death via inhibiting GSDMD.

### DSF decreases cardiac injury and improves heart function after I/R in vivo

**Fig. 4:**
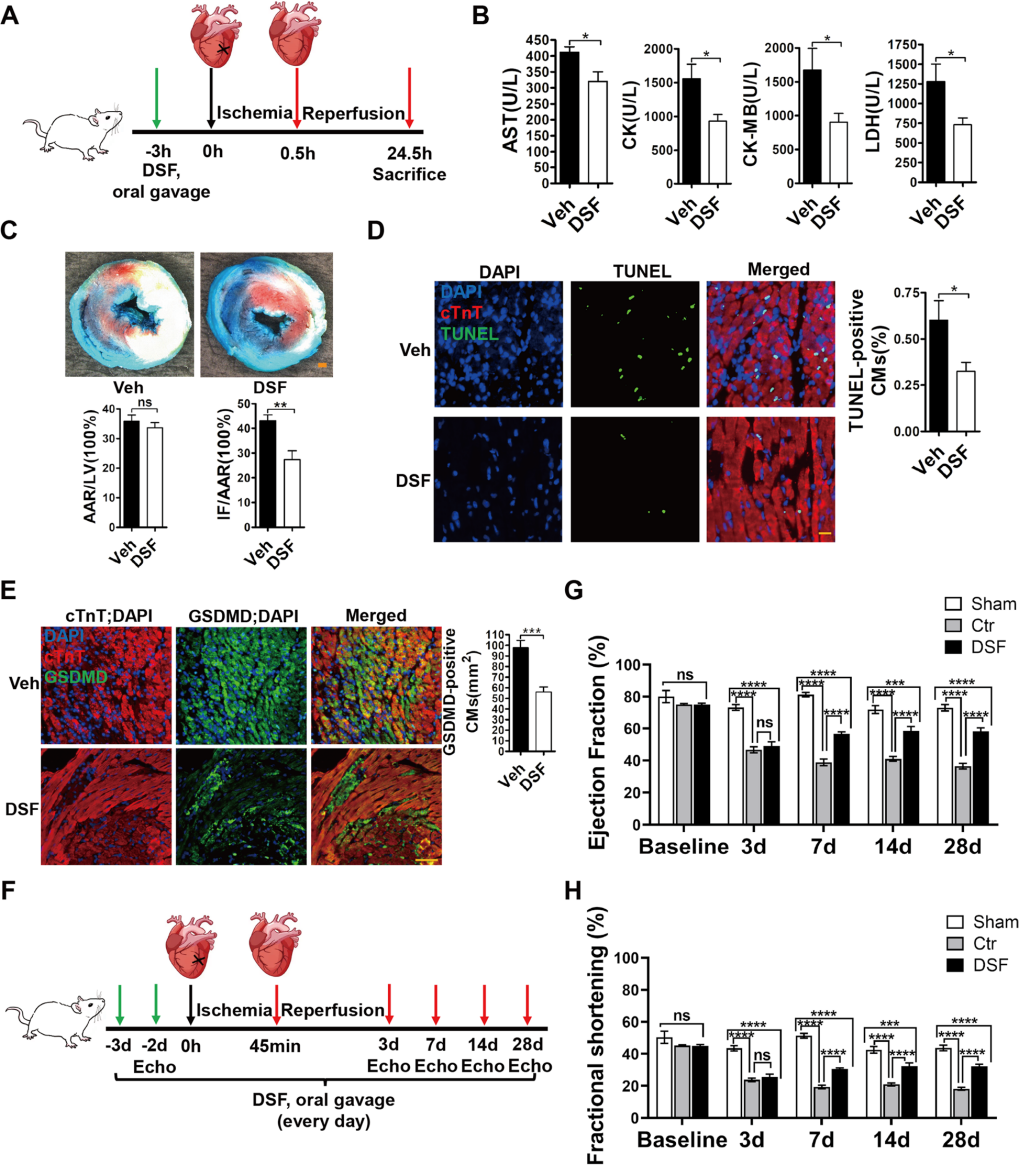
DSF decreases cardiac injury and improves heart function after I/R via inhibiting GSDMD. **A** Schematic of I/R injury of rat heart and DSF administration. **B** Evaluation of serum AST, CK, CK-MB, and LDH in rats subjected to I/R surgery with or without DSF treatment. *n* = 10 rats per group. **C** Representative images and quantitative data for infarct size (IF) and area at risk (AAR) in rat hearts subjected to I/R with or without DSF treatment. *n* = 10 rats per group. **D** Representative images and quantitative data for TUNEL staining of heart sections from rats subjected to I/R surgery. *n* = 7 rats per group. LV, left ventricle; AST, glutamic transaminase; CK, creatine kinase; CK-MB, creatine kinase isoenzyme; LDH, lactate dehydrogenase. **E** Representative images and quantitative data for GSDMD immunostaining of heart sections from rats subjected to I/R surgery. *n* = 5 rats per group. **F** Schematic of I/R injury of rat heart and DSF administration. (G and H) Serial ECHO measurements of ejection fraction (**G**), fractional shortening (**H**) of sham, control, and DSF groups after I/R. Sham, *n* = 4; control, *n* = 8; DSF, *n* = 10. Scale bar, 1 mm (**C**), 20 µm (**D**), 50 µm (**E**). *p < 0.1, **p < 0.01, ***p < 0.001, ****p < 0.0001; ns, no significant difference. Unpaired, two-tailed Student’s t-test (**B**-**E**). Two-way ANOVA followed by Tukey’s multiple comparisons test (**G** and **H**). Data are the mean ± s.e.m. (**B**-**E**, **G**, **H**)

To further test the protective effect of DSF on CMs in vivo, we administered DSF to adult male rats by oral gavage at 3 h before myocardial I/R injury (30 min ischemia/24 h reperfusion) (Fig. 4A). We found that the DSF pre-treatment group released less myocardial enzymes [serum LDH, creatine kinase (CK), creatine kinase MB isozymes (CK-MB), and aspartate aminotransferase (AST)] into the circulating blood compared with the control group (Fig. 4B), suggesting the protective role of DSF in myocardial injury. Alcian blue and triphenyl-tetrazolium chloride double staining showed that the ratio of infarct size/area at risk in DSF-pretreated mice (27.3%) was lower than that in control mice (43.2%) after I/R injury (Fig. 4C). Consistent with this, TUNEL staining data showed that DSF treatment led to less cell death in infarcted heart sections than the control group after I/R injury (Fig. 4D), and the number of GSDMD-positive CMs significantly decreased in DSF group compared with control group (Fig. 4E). These data suggested that DSF protected CMs from I/R-induced death via decreasing GSDMD expression. We next tested whether DSF treatment confers beneficial effect on heart function after I/R injury. DSF was administered to adult male rats by oral gavage once a day starting from 3 days before myocardial I/R injury (45 min ischemia/28 d reperfusion) (Fig. 4F). Echocardiography (ECHO) data showed that both ejection fraction (EF) and fractional shortening (FS) decreased significantly in the control and DSF groups compared with the sham-operated group while EF and FS were comparable in control and DSF groups at 3 days post-I/R. Importantly, DSF treatment increased both EF and FS at 7, 14 and 28 days post-I/R (Fig. 4G, H). Collectively, these results suggested that DSF effectively decreases myocardial injury and improves heart function after I/R injury via inhibiting GSDMD in vivo.

## Discussion

Although reperfusion therapy has become the standard treatment for patients with myocardial ischemia, reperfusion injury contributes to infarct size and heart failure. The complex mechanisms underlying I/R injury involve a long list of factors, in which ROS ranks at the top. The generation of ROS during reoxygenation is thought to be the consequence of dysfunction of the electron transport chain components that cannot fulfill efficient electron transfer and thus produce superoxide species (Lesnefsky et al. 2004). It has been reported that mitochondria serve as a major source of ROS as well as being a major target for ROS damage. Loss of cell viability results from mitochondrial damage that is characteristic of apoptosis, autophagy, and necrosis. A number of studies have attempted to address the mechanisms underlying each form of cell death (Hamacher-Brady 2006; Takagi et al. 2007). Undoubtedly, these modes of cell death all contribute to the final damage of infarcted hearts. Many pharmacological strategies have attempted and yet failed to attenuate reperfusion injury in phase II clinical trials, despite several promising small molecules that successfully decreased myocardial infarct size (Piot et al. 2008; Garcia-Prieto et al. 2014; Stone et al. 2012).

In this study, we identified an FDA-approved drug, DSF, as having potential for the treatment of I/R injury by inhibiting GSDMD. DSF was first discovered for its alcohol deterrent property in 1937, and then approved by the FDA for the treatment of alcoholism in 1951 due to its inhibition of ALDH (Fuller and Richard 1986). Recent investigations have identified DSF as a potent inhibitor of GSDMD pore formation, leading to reduced release of inflammatory cytokines and subsequent pyroptosis (Hu et al. 2020; Schmidt et al. 2020). Interestingly, it has been reported that CM pyroptosis triggered by myocardial I/R injury is mediated via the caspase-11-GSDMD pathway, and GSDMD deficiency decreases I/R-induced myocardial injury (Shi et al. 2021). Another study has shown that loss of GSDMD attenuates myocardial injury after AMI/IR through inhibiting GSDMD-dependent neutrophil generation and mobilization (Jiang et al. 2022). In line with these findings, DSF has been reported to prevent doxorubicin-induced cardiac dysfunction and oxidative stress in rats (Sonawane et al. 2018). The protective and anti-inflammatory effects of DSF have also been discovered in other organs, such as the inhibition of TNF-induced cell death by DSF and caspase-3-like activity in mouse liver (Zhao et al. 2000); DSF suppression of endotoxin-induced uveitis in rats (Kanai et al. 2010); DSF repression of inflammation and fibrosis in rats subjected to unilateral ureteral obstruction by inhibiting GSDMD (Zhang et al. 2021); and DSF protection by ameliorating IR-induced acute kidney injury by suppressing the caspase-11-GSDMD pathway (Cai et al. 2022). In agreement with previous findings, our work demonstrated that DSF treatment protects NRVMs from oxidative damage in vitro and infarcted hearts in vivo. In conclusion, this work supports the conclusion that DSF has great potential of becoming a new therapeutic implication for the treatment of myocardial ischemia and other ischemic diseases.

## Methods

### Animals

The neonatal (postnatal day 3, P3) and adult (8 weeks old) wild-type Sprague–Dawley rats used for this study were purchased from Vital River Laboratory Animal Technology Co., Ltd (Beijing, China). All of the male rats weighed ~ 200 g when purchased. The experimental procedures were designed in compliance with the animal protocols approved by the Institutional Animal Care and Use Committee at Peking University, Beijing, China.

### Isolation of neonatal rat ventricular myocytes (NRVMs)

The ventricles of P3 Sprague–Dawley rats were dissected and washed three times with Hanks' balanced salt solution (HBSS) without Ca^2+^ and Mg^2+^ (MacGene, CC016). The ventricles were transferred and chopped into small chunks of ~ 1 mm^3^ in 5 mL HBSS containing collagenase II (0.3 mg/mL; Gibco, 2,163,320) and trypsin (1 mg/mL; Amresco, VWRV0785) under constant stirring at 37 °C for 5 min. The supernatants were collected and 5-mL high-glucose Dulbecco's Modified Eagle Medium (DMEM; Hyclone) containing 10% fetal bovine serum (FBS) and 1% penicillin–streptomycin was added to stop digestion. This digestion process was repeated 8 to 10 times until the tissue was flocculent. All of the collected supernatants were centrifuged at 1,000 rpm for 5 min and the pellets were resuspended in high-glucose DMEM containing 10% FBS and 1% penicillin–streptomycin. The collected cells were passed through a cell strainer (100 µm, Biologix) and then seeded onto 10-cm plastic dishes for 2 h at 37 °C in a 5% CO_2_ humidified incubator. The supernatants of isolated NRVMs were then collected and centrifuged at 1,000 rpm for 5 min. The pellets were re-suspended in high-glucose DMEM containing 5% FBS, 1% penicillin–streptomycin and 20 µM cytosine arabinoside. The CMs were passed through a cell strainer and then cultured for 48 h.

### Chemical screening

The chemical screening was performed essentially as described previously (Du et al. 2022). Briefly, P3 NRVMs were isolated and cultured on 96-well plates at 15,000 cells per well. After 48 h, the culture medium was aspirated, and fresh culture medium (high-glucose DMEM containing 200 μΜ H_2_O_2_, 0.5% FBS, and 1% penicillin–streptomycin) with 2 μM small molecules was immediately applied. With regard to the preparation of 2 μM small-molecule plates, the chemical library arrayed in 384-well microplates consisted of 3,142 FDA-approved compounds (HY-L035, MedChemExpress, China); the compounds were transferred in a volume of 20 nL (10 mM) to 96-well plates using Echo 525 (Labcyte); and culture medium (100 μl DMEM containing 200 μM H_2_O_2_, 0.5% FBS, and 1% penicillin–streptomycin) was then distributed to each well using Multidrop Combi (Thermo Scientific), resulting in a final working concentration of 2 μM compounds. Both negative control (0.5% FBS) and positive control (10% FBS) were treated with 2 μM DMSO instead of compounds, while only the negative control was treated with H_2_O_2_. All of the NRVMs were then cultured for 24 h before LDH detection.

### LDH detection

After NRVMs were cultured in vitro under certain conditions, supernatants in each well (40 μL/well) were transferred to a 96-well plate, and mixed with LDH detection solution (LDH0360, Shanghai Jingyuan, China) according to the manufacturer's instructions. The LDH was measured seven times at 340 nm absorbance.

### CCK-8 assay

Before undergoing CCK-8 assay, the NRVMs were washed three times with HBSS. Fresh culture medium (high-glucose DMEM containing 0.5% FBS and 1% penicillin–streptomycin) and CCK-8 detection solution (Yeasen, 40203ES76) were applied to each well according to the manufacturer's instructions, and the cells were cultured for another 2.5 h. The CCK-8 value was measured at 450 nm absorbance. Regarding NRVMs cultured in 24-well plates, the supernatants (50 μL/well) were transferred to a 96-well plate after cells were incubated with detection solution, and the CCK-8 value was then assessed.

### Calcein AM staining

NRVMs were isolated and cultured as described above, and were first washed three times with 1 × assay buffer and were then incubated with calcein AM dye (Yeasen, 40743ES76) for 20 min following the manufacturer’s protocol. Cell viability was then visualized on Opera Phenix (Perkin Elmer).

### siRNA transfection

The ALDH1 and GSDMD siRNAs were purchased from GenePharma (Beijing). The siRNA sequences of ALDH1 and GSDMD were as follows:

- ALDH1 siRNA-1, 5′-GAUCCAUGGUCAAACAAUATT-3′;
- ALDH1 siRNA-2, 5′- GGCAGAUGUUGACAAAGCUTT-3′;
- ALDH1 siRNA-3, 5′- CACAUGGCAUCUUUAAUAATT-3′;
- GSDMD siRNA-1, 5′- CCUCCAUGAAUGUGUGUAUTT-3′;
- GSDMD siRNA-2, 5′- GGUCAAGAAUGUGAUCAAATT-3′;
- GSDMD siRNA-3, 5′- GAACCAAUGAGGAAGAAGUTT-3′.

Freshly isolated NRVMs were cultured in vitro for 48 h and were then transfected with siRNAs by transfection reagent (Lipofectamine 2000, Life Technologies) at a final siRNA concentration of 100 nM. At 6 h after transfection, the culture medium (ThermoFisher, 11,058,021) was replaced by high-glucose DMEM containing 0.5% FBS and 1% penicillin–streptomycin. At 24 h after transfection, NRVMs were exposed to 400 μM H_2_O_2_ for 30 min. The cell viability was then assessed. The knockdown efficiency of siRNAs was verified by qRT-PCR and the following oligonucleotides were used:

- ALDH1 Forward: 5’ – TTTGCGATGCCGACTTGGA – 3’;
- ALDH1 Reverse: 5’ – TTG GCTCGCTCAACACTCTT -3’;
  GSDMD Forward: 5’ – AGATCGTGGATCATGCCGTC – 3’;
- GSDMD Reverse: 5’ – TGAGTCACACGCAGCATACA – 3’.

The most effective siRNA of each gene was selected for further studies.

### ELISA

After NRVMs were exposed to H_2_O_2_ and DSF in vitro for 24 h, the supernatants in each well were centrifuged and then transferred to a 96-pore ELISA plate. Rat IL-18 was detected by using an ELISA kit according to the manufacturer's instructions (Joyee Biotechnics, JER-052).

### Western blot analysis

NRVMs from each 6-well plate were lysed with RIPA lysis buffer (Solarbio, R0010) after exposure to H_2_O_2_ and DSF for 24 h. Protein concentrations were measured using a BCA assay kit (Solarbio, PC0020). The total proteins were separated by SDS-PAGE and transferred to PVDF membranes (Millipore, IPVH00010). After blocking, the membranes were incubated overnight with anti-GAPDH (1:1000, Aksomics, KC-5G5) or anti-GSDMD/GSDMD-NT (1:1000, Abmart, PY30823S). Goat anti-rabbit IgG–HRP (1:5000, Easybio, BE0101) was used as the secondary antibody. Protein signals were detected in a ChemiDoc™ MP Imaging System (Bio-Rad).

### Ischemia–reperfusion (IR) surgery

Three hours before surgery, adult male Sprague–Dawley rats (8 weeks old) were administered DSF (50 mg/kg) by oral gavage. The rats were then anesthetized by intraperitoneal injection of tribromoethanol (300 mg/kg; Sigma-Aldrich) and ventilated on a rodent ventilator (MouseVent, Kent Scientific Corp., Torrington, CT, USA). A thoracotomy between the third and fourth ribs was performed to expose the heart. Myocardial IR was induced by tightening a slipknot around the left anterior descending coronary artery using a 6–0 nylon suture for 30 min/45 min and then loosening it (reperfusion for 24 h/28 d) before collecting the heart.

### Measurement of the serum LDH, CK, CK-MB, and AST

Rats were subjected to I/R surgery as described above. After reperfusion for 24 h, the rats were anesthetized by intraperitoneal injection of tribromoethanol (300 mg/kg). Blood samples were collected via the tail vein and kept at room temperature for 1 h prior to centrifugation at 3,000 rpm for 10 min. Serum was collected and the enzymes (LDH, CK, CK-MB and AST) were measured with four assay kits (Roche, 03004732122; Roche, 07190794190; Roche, 07190808190; Roche, 20,764,949,322) according to the manufacturer’s protocols.

### Infarct size measurement

Each rat was immediately sacrificed after blood collection as described above. The heart was dissected and washed three times with saline. The ascending aorta was cannulated and perfused with saline to further wash out the blood before the coronary artery was re-occluded at the site of occlusion. To visualize the area at risk, 1% Alcian blue (Sigma-Aldrich, A3157) was injected into the aorta. Each heart was then frozen at –20 °C for 30 min and cut into 3–4 slices from the apex to the base according to the ligation site. The slices were then incubated for 15 min at 37℃ in PBS containing 1% 2,3,5-triphenyl-tetrazolium chloride (Solarbio, G3005) to visualize the infarcted region.

### Immunostaining of heart tissues

The rat hearts from control and DSF groups were made into paraffin sections. The sections were then dewaxed in xylene, rehydrated in an ethanol series, and washed in PBS. For antigen retrieval, the sections were rinsed in pre-heated citric acid buffer (pH 9.5) and boiled in a microwave for 15 min. After washing in PBS, the sections were blocked in 10% FBS in PBST for 30 min. TUNEL staining on paraffin sections was performed with an assay kit (Beyotime, C1088) according to the manufacturer’s protocol. The sections were then incubated overnight at 4 °C with the primary antibodies: anti-GSDMD/GSDMD-NT antibody (1:100, Abmart, PY30823S) and anti-Cardiac Troponin T antibody (1:300, Abcam, ab8295). After three washes with PBS for 5 min each, the sections were incubated with the corresponding fluorescence-labeled secondary antibodies: Donkey to Rabbit IgG—H&L (Abcam, ab150073) and Donkey to Mouse IgG—H&L (Abcam, ab150110) at room temperature for 2 h, and counterstained with DAPI. Dead CMs (green fluorescence) in the left ventricles were counted. GSDMD-positive CMs (green fluorescence) were counted in the infarct border zone and infarct zone of left ventricles.

### Echocardiography (ECHO)

ECHO was performed on rats anesthetized with 1% isoflurane at 2 days before I/R, and at 3, 7, 14, and 28 days after I/R. A high-resolution Vevo 2100 Ultrasound (Visual Sonics) equipped with a 20-MHz variable frequency transducer was used to measure heart function. ECHO data were acquired using M-mode. Data were acquired and analyzed by an investigator who was blinded to the drug treatments of the animal groups.

### Statistical analysis

All of the results are presented as the mean ± standard error of the mean (SEM). Statistical analysis was performed with Graphpad Prism 8.3.0. Two groups were statistically compared using the unpaired Student’s t-test with two-tailed *P* values. Comparisons between multiple groups were determined by one-way ANOVA with Dunnett’s test.

## Supplementary Information

**Additional file 1:** **Fig. S1.** DSF treatment decreases H_2_O_2_-induced CM injury in a dose-dependent manner.

## Data Availability

The datasets used and/or analyzed during the current study are available from the corresponding author Prof. Jing-Wei Xiong upon requests.

## Abbreviations

DSF: Disulfiram
GSDMD: Gasdermin D
AMI: Acute myocardial infarction
I/R: Ischemia/reperfusion
ROS: Reactive oxygen species
NRVMs: Neonatal rat ventricular myocytes
CM: Cardiomyocyte
ALDH: Acetaldehyde dehydrogenase
AST: Glutamic transaminase
CK: Creatine kinase
CK-MB: Creatine kinase MB isoenzymes
LDH: Lactate dehydrogenase
EF: Ejection fraction
FS: Fractional shortening
ECHO: Echocardiography
