# Supplementary figures and images for: Chemical screening links disulfiram with cardiac protection after ischemic injury
Source: Cell Regen. 2023 Jul 19;12:25. doi: 10.1186/s13619-023-00170-x (PMC10356704; doi:10.1186/s13619-023-00170-x)

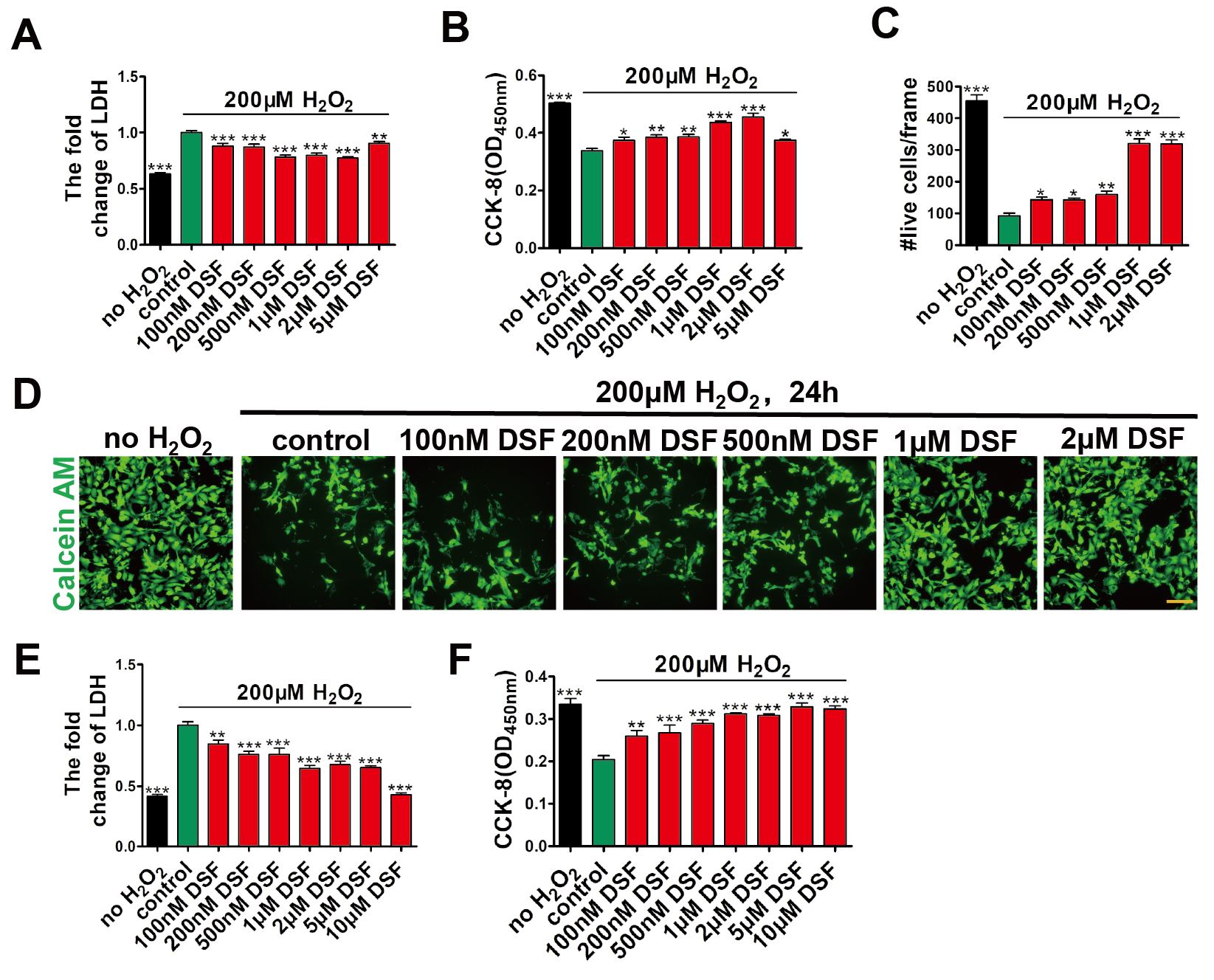

Supplement: Supplementary file 1 — Additional file 1: Fig. S1. DSF treatment decreases H2O2-induced CM injury in a dose-dependent manner. [file 13619_2023_170_MOESM1_ESM.tif]
